# Neurodegenerative mortality among National Football League Players

**DOI:** 10.1016/j.eclinm.2026.104051

**Published:** 2026-07-08

**Authors:** Charlotte B. Luster, Bobak Abdolmohammadi, Michael J. Mastrodicasa, Christopher J. Nowinski, Evan D. Feigel, Brenna Finegan, Adam J. White, Eric J. Connors, Craig A. Rovito, Ross D. Zafonte, Michael L. Alosco, Ann C. McKee, Jesse Mez, Daniel H. Daneshvar

**Affiliations:** a Department of Physical Medicine and Rehabilitation, Spaulding Rehabilitation Hospital, Boston, MA, USA; b Boston University Alzheimer's Disease and CTE Centers, Boston University Chobanian & Avedisian School of Medicine, Boston, MA, USA; c Concussion & CTE Foundation, Boston, MA, USA; d Department of Physical Medicine and Rehabilitation, Harvard Medical School, Boston, MA, USA; e Department of Physical Medicine and Rehabilitation, Massachusetts General Hospital, Boston, MA, USA; f Department of Physical Medicine Rehabilitation and Anesthesiology, University of Missouri School of Medicine, Columbia, MO, USA; g Department of Neurology, Boston University Chobanian & Avedisian School of Medicine, Boston, MA, USA; h Framingham Heart Study, Boston University School of Medicine, Boston, MA, USA; i Professional Footballers Association, England, UK

**Keywords:** Neurodegenerative Disease, Epidemiology

## Abstract

**Background:**

Empirical research demonstrates elevated neurodegenerative mortality among individuals with repetitive head impact (RHI) exposure, including National Football League (NFL) players. This investigation addressed prior methodological limitations, including selection bias, subjective diagnoses, and retrospective reporting, by analyzing the relationship between RHI exposure and neurodegenerative mortality in a fully enumerated, 5.8-fold larger cohort of NFL players.

**Methods:**

A population-based retrospective cohort study was conducted comprising all current and former NFL athletes who debuted between 1960 and 2019 and played at least one regular or postseason NFL game, with National Death Index records (1979–2023) matched to Sports Reference, LLC data. Standardized mortality ratios (SMRs) were calculated from National Institute for Occupational Safety and Health data compared to an age-, sex-, race-, and calendar-year-standardized general population. Sensitivity analysis assessed whether the observed excess neurodegenerative mortality could be attributed to competing risks using a cause-specific hazard simulation.

**Findings:**

A total of 19,824 athletes had a cumulative 518,833 person-years (mean = 26.2 years, SD = 16.2), with 1994 decedents. NFL players exhibited lower all-cause mortality (SMR = 0.70; 95% CI: 0.67–0.74) but higher neurodegenerative mortality (SMR = 3.94; 95% CI: 3.38–4.56), including amyotrophic lateral sclerosis (SMR = 4.55; 95% CI = 3.13–6.38), all-cause dementia (SMR = 3.80; 95% CI = 3.11–4.60), and Parkinson's disease (SMR = 3.88; 95% CI: 2.76–5.30). Cause-specific hazard simulation indicated that competing risks alone would inflate the expected NDD SMR by a factor of 1.30, yielding a residual neurodegenerative SMR of 3.04 (95% CI: 2.63–3.50).

**Interpretation:**

Neurodegenerative mortality was nearly four times higher in NFL players compared to the general population and remained threefold higher after accounting for competing risks. Together, these findings strengthen the evidence for RHI exposure-related neurodegenerative mortality in NFL players that cannot be explained by differential survivorship.

**Funding:**

The National Institute of Neurological Disorders and Stroke [U54NS115266; U01NS086659], the National Institute on Aging [P30AG13846; P30AG072978], and the Maloney/Carpenter Trauma-Related Neurodegenerative Disease Research Fund.

**Research in context**

**Evidence before this study**

We searched PubMed for peer-reviewed studies examining neurodegenerative mortality among individuals with high exposure to repetitive head impacts (RHI). Searches were conducted from database inception to January 15, 2026. Search terms included combinations of “National Football League”, “American football”, “repetitive head impacts”, “neurodegenerative disease”, “amyotrophic lateral sclerosis,” “dementia,” “Parkinson's disease,” “Alzheimer's disease”, and “mortality.” Existing epidemiologic studies consistently report elevated neurodegenerative mortality among NFL players, despite evidence of lower overall mortality compared to the general population. However, prior studies have been limited by sample size, selection bias, and competing mortality risks, which may bias estimates of neurodegenerative disease risk.

**Added value of this study**

This investigation addressed prior methodological limitations, including selection bias, subjective diagnoses, and retrospective reporting, by analyzing the relationship between RHI exposure and neurodegenerative mortality in a fully enumerated, 5.8-fold larger cohort of NFL players. In the largest study of mortality in NFL players to date, robust internal comparisons and competing-risk simulations enabled more accurate examination of the relationship between cumulative RHI exposure and neurodegenerative mortality risk. Accounting for survivorship benefits, NFL players had a threefold greater risk of neurodegenerative mortality.

**Implications of all the available evidence**

These findings strengthen evidence of RHI exposure-related neurodegenerative mortality in NFL players, an effect not explained by survivorship advantages associated with elite athletic selection. Together, this body of evidence supports the evaluation of healthspan-focused interventions and reinforces the need for policies limiting RHI exposure among NFL players.

## Introduction

Neurodegenerative disease (NDD) mortality among individuals with high exposure to repetitive head impacts (RHI) poses a critical public health concern given the high proportion of individuals who experience RHI through sport, occupation, and other sources.^1^ Recent research efforts assessing NDD mortality has centered on elite athlete populations, including National Football League (NFL) players, as they represent a fully enumerated cohort with well-characterized RHI exposure and mortality data allowing for a comprehensive evaluation of risk factors associated with NFL participation.^2^ Mounting evidence demonstrates that RHI sustained during American football is associated with increased risk for NDD, including Alzheimer's disease (AD)/dementia, amyotrophic lateral sclerosis (ALS), chronic traumatic encephalopathy (CTE), and Parkinson's disease (PD).^1^, ^2^, ^3^, ^4^ Neuropathological investigations report that cumulative RHI is associated with progressive tauopathy, axonal injury, and neuroinflammation, which may accelerate neurodegenerative processes.^1^

Early epidemiological evidence of NDD among NFL players reported an approximately three-fold increased risk of mortality in players with at least five seasons professionally, and higher mortality rates from AD/dementia and ALS, and among those in speed positions.^3^ However, subtype- and position-specific mortality rates were found only to be significant when examining multiple causes of death.^3^ A recent investigation reported that a subset of these NFL players had a three-fold higher neurodegenerative mortality rate than Major League Baseball (MLB) players without subtype-specific elevations in neurodegenerative underlying causes of death.^2^ Notably, this absence of subtype-specific NDD mortality is reflective of methodological constraints, including small sample sizes and insufficient person-year accumulations.^2^^,^^3^ However, studies addressing these limitations reported a four-fold increased risk of ALS mortality among NFL players with nonsignificant elevations among Black players compared to their White counterparts.^4^ Although not previously evaluated in mortality analyses specifically among NFL players, general population data suggest non-White individuals have a higher prevalence of dementia.^5^ Additionally, earlier onset NDD has been demonstrated among NFL players.^6^

Previous studies of former professional athletes also identified evidence of NDD-excluded survivor benefits.^2^^,^^3^ For example, investigations of elite athletes identified lower all-cause mortality in NFL players compared to the general population, including a lower proportion of deaths from cancer, cardiovascular disease, injury, violence, and all other causes.^3^ These differences may reflect a selection or sports training variant of the healthy worker survivor effect, where healthier individuals remain employed longer and therefore were observed to be relatively healthier compared to unemployed and shorter employed individuals.^7^ Other investigations accounted for survivorship effects by comparing mortality rates across professional sports, and observed elevated all-cause, neurodegenerative, and cardiovascular disease mortality in former NFL athletes compared to former MLB athletes.^2^ Although an important finding, this comparison may not fully account for these effects as longer average career lengths in MLB compared to NFL athletes could result in survival bias, which may be further compounded by demographic differences between the leagues conferring distinct baseline health risk profiles and results in residual confounding.^2^^,^^7^ Internal comparisons among subgroups with different degrees of exposure and increased latencies may provide an alternate means of minimizing survivor effects.^7^

The relationship between RHI and NDD remains the subject of extensive debate.^8^, ^9^, ^10^, ^11^, ^12^ Studies reporting increased NDD risk among those with substantial RHI exposure have been criticized for methodological and conceptual limitations.^8^, ^9^, ^10^, ^11^, ^12^ Notably, all neuropathological studies are subject to selection bias from the non-random nature of brain donation that may overrepresent symptomatic individuals and inflate perceived prevalence of rare conditions.^8^, ^9^, ^10^, ^11^ Additionally, previous literature assessing the link between NDD and RHI are often published by select groups, which some maintain overly rely on retrospective informant interviews and postmortem evaluations, obscuring the temporal and causal link between exposure and symptomatology.^10^^,^^12^ Finally, some suggest the relationship between RHI and NDD has been publicized prematurely with overreaching, fear-inducing statements that exceed what the data support.^10^^,^^12^

Our study aims to address these concerns in a manner not previously achieved in the literature, utilizing a 5.8-fold larger,^2^^,^^3^ fully enumerated cohort of NFL players to better characterize neurodegenerative risks associated with American football participation. Leveraging National Death Index (NDI) records for all athletes who played in a single NFL game from 1960 to 2019, we avoid selection bias, subjective diagnostic determinations, and reliance on retrospective reports by expanding outcome assessment to an unselected group of physicians, namely those who signed the death certificates. Our larger sample, including 8- to 17.8-fold more decedents with NDD underlying causes of death than previous work,^2^^,^^3^ may provide better insight into the relative disease burden and allow for more robust sub-analyses exploring differences in factors such as age and duration of play. We assessed neurodegenerative mortality by calculating standardized mortality ratios (SMRs) relative to the general population, and compared SMRs by race, position, career duration, and age to identify subgroups most at-risk. We hypothesized that (1) NFL players would have higher overall neurodegenerative, all-cause dementia, ALS, and PD mortality,^2^^,^^3^ (2) that speed positions (all non-linemen),^2^^,^^3^ non-White race,^4^^,^^5^ longer career duration,^2^^,^^3^ and younger ages would confer particular risk for neurodegenerative mortality,^6^ and (3) NFL players would have NDD-excluded survivor benefits.^3^

## Methods

### Study population

**Fig. 1:**
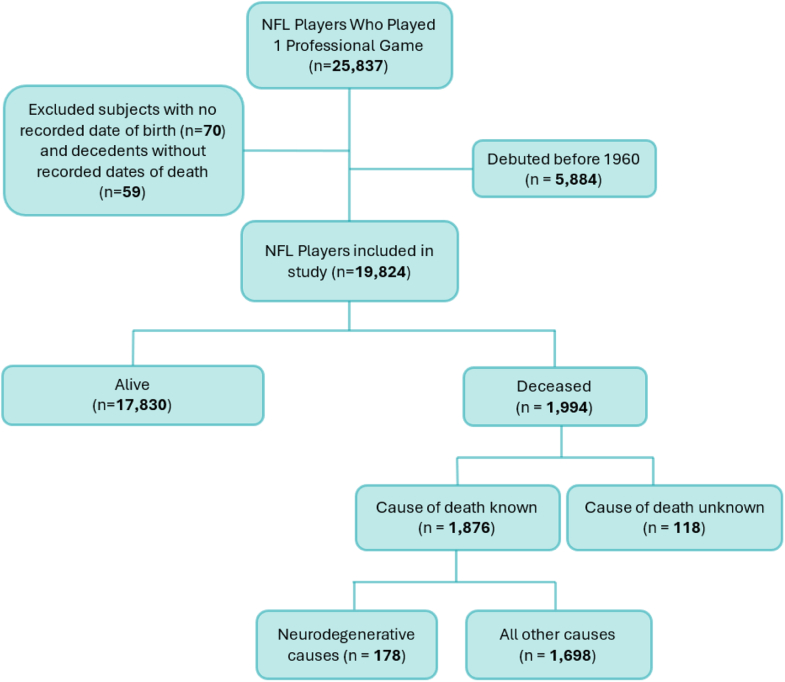
Flowchart of study inclusion.

This study utilized the Sports Reference, LLC database which contains information on all NFL players since the league's founding in 1920 that is licensed for use by leading sports organizations and leveraged by researchers.^4^ The Sports Reference, LLC database includes date of birth, date of death, location of birth (city, state, country), race, NFL games played, NFL games started, NFL draft pick number, height at NFL debut, weight at NFL debut, duration of NFL career, number of NFL Pro Bowl appearances, NFL Hall of Fame status, and NFL position played for all NFL players. The database contains data on both the NFL and the American Football League (AFL). Prior to the NFL and AFL merger in 1966, many athletes played in both leagues and this study considers players from both leagues NFL players. NFL players who debuted between 1960 and 2019 with ≥1 regular or postseason game were included for analysis (Fig. 1), with 6013 players excluded due to debut year or incomplete data. Playing positions were grouped into twelve categories: defensive back (cornerback, halfback, safety, nickelback), defensive line (defensive tackle, nose tackle, defensive end, edge rusher), linebacker, offensive line (guard, offensive tackle, center), quarterback, running back (halfback, tailback, blocking back, fullback, wing back), kicker, punter, tight end, wide receiver (split end, slot back, and flanker), and multiple (end or more than one position listed in the database). Positions were further categorized as non-speed (offensive and defensive linemen) and speed (all other positions). NDI records were matched to the cohort with recorded deaths between 1979 (when NDI began)^13^ and 2023. NDI-Plus metrics were applied to verify matches between databases with matches ≥ Class 3 considered true matches.^13^ Underlying (primary) cause of death codes were extracted from NDI death certificate data.

### Statistical analysis

All analyses were conducted using R Software v4.5.2. Unadjusted means and frequencies were generated for demographic and exposure characteristics. Differences between decedents by cause of death were examined by Two-Sample t-test, Welch Two-Sample t-test, chi-square, or Fisher's Exact test. Equality of variances across groups was assessed using Levene's test. Where variances were equal, the standard two-sample t-test was applied; where equality of variances could not be assumed, the Welch two-sample t-test was used. For categorical variables, chi-square tests were applied when expected cell frequencies were ≥5 in all cells; Fisher's Exact test was used when expected cell frequencies fell below this threshold.

The National Institute for Occupational Safety and Health (NIOSH) Life Table Analysis System R package, *LTASR*,^14^ was used to calculate SMRs and confidence intervals (CI) to compare mortality rates of the cohort to an age, sex, race, and calendar year standardized general population (Supplemental Table S1). NIOSH originally created the Life Table Analysis System (LTAS) or LTAS.NET in the 1970's to help epidemiologists evaluate incidence and mortality in occupational cohort studies.^3^ NIOSH and the CDC deprecated LTAS.NET in 2022 and announced the launch of the R package *LTASR*.^14^ This package allows researchers to calculate SMRs from cohorts with complete data on sex, race, date of birth, date to begin follow-up, date late observed, vitality status, ICD code of death, and ICD revision number. *LTASR* contains Centers for Disease Control and Prevention (CDC) rate files with mortality rates for the U.S. general population stratified by age (5-year intervals, 15–85+), sex, race (White/non-White), calendar year (5-year periods, 1960–2023), and cause of death coded using ICD-7, ICD-8, ICD-9, and ICD-10.^14^ The *LTASR* package employs indirect standardization, wherein each cohort member contributes person-time to strata defined by their individual age, sex, race, and calendar year categories. Expected deaths are calculated by multiplying the stratum-specific person-time by the corresponding U.S. general population mortality rates derived from CDC vital statistics rate files. To date, *LTASR* provides one rate file with U.S. general population mortality rates for 119 pre-determined “minor” causes of death which map to 28 “major” causes of death. These groupings categorize neurodegenerative diseases, including dementia, Alzheimer's disease, Parkinson's disease, and amyotrophic lateral sclerosis as “other diseases of the nervous system”. The study team obtained a neurodegenerative disease rate file from NIOSH for the purpose of the present study which contained the three pre-determined neurodegenerative cause groupings detailed in Supplemental Table S1. The rate file pre-loaded in LTASR and the neurodegenerative rate file only have mortality rate data for underlying (primary) cause of death. Multiple (secondary) causes of death rates are not currently available. Consequently, analyses were limited to the underlying cause of death code listed on the death certificate. Subjects with unknown cause of death (n = 118; 5.9%) lacked a match in the NDI but were known deceased.

Person-year accumulation started June 15th of debut year until death or last follow-up. *LTASR* does not currently support evaluation of SMR differences by continuous variables (e.g. exposure). Therefore, continuous variables of interest, including career duration and age, were dichotomized. Secondary analysis calculated standardized rate ratios (SRRs) and 95% CIs to compare mortality by race (White = internal referent), position (non-speed = internal referent), career duration (1–4 years = internal referent), and age (≥60-years-old = internal referent) among NFL players. SRRs were calculated manually as the ratio of two subgroup-specific SMRs generated by *LTASR*. Confidence intervals for the SRR were derived on the log scale using the formula SE (log SRR) = √(1/O_1_ + 1/O_2_), where O_1_ and O_2_ are the observed death counts in each subgroup, with back-transformation to obtain the 95% CI.^15^ Positional and career duration categories were chosen to align with previous literature^2^^,^^3^ and the age at death cutoff was based on the mean age at death of the cohort. Additional analyses further evaluated NDD mortality difference by age (<50 and ≥50) to investigate alignment with previous work.^16^ To examine age-based patterns of cause-specific mortality, the cohort was stratified into five-year age intervals and *LTASR* SMR calculations were applied to each stratum separately, yielding expected deaths by cause and age group.

Sensitivity analysis assessed whether the observed excess neurodegenerative mortality could be attributed to competing risks using a cause-specific hazard simulation.^17^ We constructed a null counterfactual model in which NFL players were assigned the general population neurodegenerative mortality rate at each age group (5-year intervals) while retaining their observed lower mortality rates for all competing causes (cardiovascular disease, cancer, injury, suicide, other causes). In the null model, survival to each age group was estimated using the NFL-specific total hazard across all causes. Expected neurodegenerative deaths were then calculated by applying the general population neurodegenerative mortality rate to the NFL survival curve. The ratio of null model to standard LTASR expected neurodegenerative deaths defined the competing risks inflation factor, representing SMR elevation attributable solely to differential survivorship. Uncertainty was quantified using 1000 Poisson bootstrap samples, with 95% CIs derived from the 2.5th and 97.5th percentiles. An additional tipping point analysis was conducted in which all competing cause mortality rates were scaled simultaneously from their observed values to zero. The residual neurodegenerative SMR and 95% CI were estimated at each scalar value to identify the competing mortality assumption under which the excess neurodegenerative mortality would no longer be statistically significant. Significance was determined α = .05. Additional methods are detailed in Supplemental Methods.

### Ethics

This study was approved by Boston University Medical Center institutional review board, followed the Strengthening the Reporting of Observational Studies in Epidemiology reporting guidelines, and adhered to the Declaration of Helsinki. All participants were decedents and thus informed consent was not required or obtained.

### Role of funding source

The funders had no role in the design and conduct of the study; collection, management, analysis, and interpretation of the data; preparation, review, or approval of the manuscript; and decision to submit the manuscript for publication.

## Results

A total of 19,824 athletes had a cumulative 518,833 person-years (mean = 26.2 years, SD = 16.2). Among 1994 decedents, 178 died of NDD, including 106 from all-cause dementia, 33 from ALS, and 39 from PD (Table 1). NFL players who died of NDD were older (+13.4 years; 95% CI: 11.7–15.3), more likely White (+18.4%; p < 0.001), had lower BMIs while playing (−1.4 kg/m^2^; 95% CI: (−0.9)−(−1.8)), had longer careers (+0.84 years; 95% CI: 0.2–1.4), played in more games (+11.4 games; 95% CI: 3.1–19.7), started in more games (+7.2 games, 95% CI: 0.02–14.4), and attended more Pro Bowls (+0.14 Pro Bowls; 95% CI: 0.004–0.27) than those who died of all other causes (Table 1). White decedents were older than non-White decedents (+8.9 years; 95% CI: 7.6–10.2) and decedents with careers ≥5 seasons were older than decedents with careers <5 seasons (+3.7 years; 95% CI: 2.4–5.1). Non-White players made up 70% (n = 7/10) of ALS decedents <50-years-old, 55% (n = 11/20) of ALS decedents <60-years-old, 100% (n = 1) of dementia decedents <50-years-old, and 67% (n = 2/3) of dementia decedents <60-years-old (Supplemental Table S2). Age was similar between speed and non-speed decedents (+1.1 years; p = .201). Non-White players had longer careers (+0.39 years; 95% CI: 0.05–0.73) and were more likely to play in speed positions (+4.7%; p = .012) than White players.

**Table 1:** Characteristics of National Football League Players (NFL) by death certificate neurodegenerative cause of death.

Characteristics	All decedents (n = 1994)	All-cause dementia (n = 106)	ALS (n = 33)	PD (n = 39)	Neurodegenerative (n = 178)	Non-neurodegenerative (n = 1816)	p-value
Mean age at death (SD)	60.3 (15.6)	76.0 (6.86)	56.9 (12.3)	76.5 (6.78)	72.5 (11.0)	59.1 (15.5)	<0.001
Race (%)							<0.001
Non-white	938 (47.0%)	29 (27.4%)	15 (45.5%)	10 (25.6%)	54 (30.3%)	884 (48.7%)	<0.001
White	1056 (53.0%)	77 (72.6%)	18 (54.5%)	29 (74.4%)	124 (69.7%)	932 (51.3%)	<0.001
Sex (%)							
Male	1994 (100%)	106 (100%)	33 (100%)	39 (100%)	178 (100%)	1816 (100%)	
Mean height in inches (SD)	74.0 (2.27)	73.4 (2.06)	73.6 (1.73)	74.0 (1.75)	73.6 (1.95)	74.1 (2.29)	0.001
Mean weight in pounds (SD)	230 (33.3)	216 (26.4)	218 (28.6)	218 (25.4)	217 (26.5)	231 (33.7)	<0.001
Mean BMI in kg/m^2^ (SD)	29.4 (3.34)	28.1 (2.70)	28.3 (3.14)	28.0 (2.63)	28.1 (2.76)	29.5 (3.37)	<0.001
Position (%)							0.079
Defensive back	270 (13.5%)	20 (18.9%)	9 (27.3%)	3 (7.7%)	32 (18.0%)	238 (13.1%)	
Defensive lineman	202 (10.1%)	6 (5.7%)	2 (6.1%)	1 (2.6%)	9 (5.1%)	193 (10.6%)	
Kicker	28 (1.4%)	2 (1.9%)	0 (0%)	1 (2.6%)	3 (1.7%)	25 (1.4%)	
Linebacker	234 (11.7%)	17 (16.0%)	3 (9.1%)	2 (5.1%)	22 (12.4%)	212 (11.7%)	
Offensive lineman	247 (12.4%)	6 (5.7%)	2 (6.1%)	7 (17.9%)	15 (8.4%)	232 (12.8%)	
Punter	24 (1.2%)	1 (0.9%)	0 (0%)	1 (2.6%)	2 (1.1%)	22 (1.2%)	
Quarterback	70 (3.5%)	7 (6.6%)	2 (6.1%)	2 (5.1%)	11 (6.2%)	59 (3.2%)	
Running back	277 (13.9%)	19 (17.9%)	3 (9.1%)	5 (12.8%)	27 (15.2%)	250 (13.8%)	
Tight end	85 (4.3%)	3 (2.8%)	0 (0%)	3 (7.7%)	6 (3.4%)	79 (4.4%)	
Wide receiver	98 (4.9%)	2 (1.9%)	1 (3.0%)	3 (7.7%)	6 (3.4%)	92 (5.1%)	
Multiple	457 (22.9%)	23 (21.7%)	11 (33.3%)	11 (28.2%)	45 (25.3%)	412 (22.7%)	
Unknown	2 (0.1%)	0 (0%)	0 (0%)	0 (0%)	0 (0%)	2 (0.1%)	
Exposure characteristics							
Mean years in NFL (SD)	5.35 (3.88)	6.23 (3.97)	5.30 (4.10)	6.51 (4.48)	6.12 (4.11)	5.28 (3.85)	0.006
Mean NFL games played (SD)	63.0 (54.1)	74.2 (54.5)	64.4 (58.9)	78.7 (63.2)	73.4 (57.2)	62.0 (53.7)	0.007
Mean NFL games started (SD)	37.0 (46.8)	45.1 (48.1)	40.0 (47.3)	42.4 (56.0)	43.5 (49.6)	36.3 (46.4)	0.0495
Pro bowl, ever (%)	292 (14.6%)	23 (21.7%)	2 (6.1%)	11 (28.2%)	36 (20.2%)	256 (14.1%)	0.036
Mean pro bowls (SD)	0.260 (0.753)	0.434 (0.956)	0.0909 (0.384)	0.513 (0.942)	0.388 (0.884)	0.248 (0.738)	0.042
Hall of fame (%)	38 (1.9%)	3 (2.8%)	0 (0%)	1 (2.6%)	4 (2.2%)	34 (1.9%)	0.771

Percentages represent the number divided by all players with that cause of death. Chi-square and Fisher's exact test were used to assess differences in categorical variables by neurodegenerative vs non-neurodegenerative cause of death. T-test was used to assess differences in continuous variables by neurodegenerative vs non-neurodegenerative cause of death. Height, weight, and BMI were measured at NFL debut. SD = standard deviation, BMI = body mass index, kg = kilogram, m = meter.

**Fig. 2:**
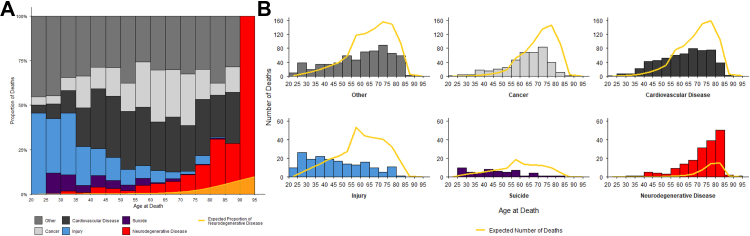
(A) Relative proportion of each cause of death by age at death and expected proportion of neurodegenerative disease. (B) Observed and expected number of deaths for each cause by age at death. Expected deaths derived from LTASR based on an age, sex, race, and calendar year standardized sample.

Players had reduced all-cause (SMR = 0.70; 95% CI: 0.67–0.74), cancer (SMR = 0.64; 95% CI: 0.58–0.71), cardiovascular disease (SMR = 0.74; 95% CI: 0.68–0.81), injury (SMR = 0.56; 95% CI: 0.48–0.65), suicide (SMR = 0.50; 95% CI: 0.38–0.66), and other cause (SMR = 0.65; 95% CI: 0.60–0.70) mortality but increased neurodegenerative mortality (SMR = 3.94; 95% CI: 3.38–4.56), relative to an age-, sex-, race-, and calendar-year-standardized general population (Table 2). Fig. 2 shows the proportion, number, and expected deaths for each cause of death category and Supplemental Table S3 reports the sample size per age group. Mortality was higher for ALS (SMR = 4.55; 95% CI = 3.13–6.38), all-cause dementia (SMR = 3.80; 95% CI = 3.11–4.60), and PD (SMR = 3.88; 95% CI: 2.76–5.30) compared to the general population (Table 3).

**Table 2:** All-cause mortality in National Football League Players.

Underlying cause of death	Observed	Expected	Excess	SMR	95% CI
Cancer	405	631.66	−226.66	**0.64**	0.58–0.71
Cardiovascular disease	568	762.81	−194.81	**0.74**	0.68–0.81
Injury	183	327.09	−144.09	**0.56**	0.48–0.65
Neurodegenerative disease	178	45.21	132.79	**3.94**	3.38–4.56
Suicide	53	105.09	−52.09	**0.50**	0.38–0.66
Other	607	939.09	−332.09	**0.65**	0.60–0.70
**Overall**	1994	2829.62	−835.62	**0.70**	0.67–0.74

Standardized Mortality Ratio (SMR) is standardized by age at death, calendar year of death, sex, and race. Excess deaths represent the observed deaths minus the expected deaths. CI = confidence interval. Bold indicates p < 0.05.

**Table 3:** Neurodegenerative mortality in National Football League Players.

Underlying Cause of Death	Observed	Expected	Excess	SMR	95% CI
All-cause Dementia	106	27.89	78.11	**3.80**	3.11–4.60
Amyotrophic Lateral Sclerosis	33	7.26	25.74	**4.55**	3.13–6.38
Parkinson's Disease	39	10.06	28.94	**3.88**	2.76–5.30
**Overall**	178	45.21	132.79	**3.94**	3.38–4.56

Standardized Mortality Ratio (SMR) is standardized by age at death, calendar year of death, sex, and race. Excess deaths represent the observed deaths minus the expected deaths. CI = confidence interval. Bold indicates p < 0.05.

Secondary analyses showed that speed players had higher neurodegenerative (SRR = 1.67, 95% CI: 1.09–2.57) and all-cause dementia mortality (SRR = 2.04; 95% CI: 1.12–3.71) than non-speed players, without positional differences in ALS or PD mortality (Table 4). Non-White players had higher ALS mortality than White players (SRR = 2.26; 95% CI: 1.14–4.49), but no racial differences were observed for other NDDs. Players with careers ≥5 years had higher neurodegenerative (SRR = 1.86, 95% CI: 1.38–2.51), all-cause dementia (SRR = 1.99; 95% CI: 1.34–2.95), and PD (SRR = 2.38; 95% CI: 1.23–4.65) mortality compared to players with careers <5 years. Players <60-years-old had a 3.5-fold (95% CI: 1.11–10.90) increased neurodegenerative mortality than players ≥60 and higher ALS (SMR = 15.38; 95% CI: 9.40–23.76) and all-cause dementia mortality (SMR = 7.69; 95% CI: 1.59–22.48) compared to an age-standardized general population. Additional analysis revealed players <50-years-old had a 41.7-fold (95% CI: 19.98–76.63) increased risk of ALS mortality compared to those ≥50. However all-cause dementia and PD mortality was not significantly higher among those under 50-years-old (Supplemental Table S4). Cause-specific hazard simulation indicated that competing risks alone would inflate the expected NDD SMR by a factor of 1.30, yielding a residual neurodegenerative SMR of 3.04 (95% CI: 2.63–3.50). The residual neurodegenerative SMR remained significant (SMR = 1.71; 95% CI: 1.47–1.96) under the assumption of zero mortality from all competing causes.

**Table 4:** Positional, racial, career duration, and age differences in neurodegenerative mortality in National Football League Players.

Underlying cause of death	Speed position					Non-speed position					Speed vs Non-speed: SRR (95% CI)
	Observed	Expected	Excess	SMR	95% CI	Observed	Expected	Excess	SMR	95% CI	
ALS	29	5.77	23.23	**4.99**	3.34–7.01	4	1.49	2.51	2.76	0.75–7.06	1.81 (0.64–5.15)
All-cause dementia	94	22.13	71.87	**4.25**	3.45–5.24	13	6.15	6.85	**2.09**	1.13–3.61	**2.04** (1.12–3.71)
Parkinson's disease	31	7.80	32.20	**3.91**	1.66–5.55	8	2.13	5.87	**3.76**	1.62–7.40	1.04 (0.48–2.26)
Overall	154	35.30	118.70	**4.29**	3.64–5.03	24	9.33	14.67	**2.57**	1.65–3.83	**1.67** (1.09–2.57)

Underlying cause of death	Non-white					White					Non-white vs white: SRR (95% CI)
	Observed	Expected	Excess	SMR	95% CI	Observed	Expected	Excess	SMR	95% CI	
ALS	15	1.95	13.05	**7.69**	4.31–12.69	18	5.30	12.70	**3.40**	2.01–5.37	**2.26** (1.14–4.49)
All-cause dementia	29	8.20	20.80	**3.54**	2.37–5.08	77	19.68	57.32	**3.91**	3.09–4.89	0.90 (0.59–1.39)
Parkinson's disease	10	2.04	7.96	**4.90**	2.35–9.01	29	8.02	20.98	**3.62**	2.42–5.19	1.36 (0.66–2.78)
Overall	54	12.19	41.81	**4.43**	3.33–5.78	124	33.00	91.00	**3.76**	3.12–4.48	1.18 (0.86–1.63)

Underlying cause of death	5+ NFL seasons played					1–4 NFL seasons played					5+ vs 1–4 Seasons: SRR (95% CI)
	Observed	Expected	Excess	SMR	95% CI	Observed	Expected	Excess	SMR	95% CI	
ALS	16	3.28	12.72	**4.88**	2.79–7.92	17	3.98	13.02	**4.27**	2.49–6.84	1.14 (0.58–2.26)
All-cause dementia	66	12.64	53.36	**5.22**	4.04–6.64	40	15.24	24.76	**2.62**	1.87–3.57	**1.99** (1.34–2.95)
Parkinson's disease	26	4.58	21.42	**5.68**	3.71–8.32	13	5.47	7.53	**2.38**	1.27–4.06	**2.38** (1.23–4.65)
Overall	108	20.50	87.50	**5.27**	4.32–6.36	70	24.69	45.31	**2.21**	2.21–3.58	**1.86** (1.38–2.51)

Underlying cause of death	Under age 60 at death					Age 60+ at death					Under vs over 60: SRR (95% CI)
	Observed	Expected	Excess	SMR	95% CI	Observed	Expected	Excess	SMR	95% CI	
ALS	20	1.30	18.70	**15.38**	9.40–23.76	13	5.96	7.04	**2.18**	2.08–4.92	**7.05** (3.51–14.18)
All-cause dementia	3	0.39	2.61	**7.69**	1.59–22.48	103	27.49	75.51	**3.74**	1.16–3.73	2.05 (0.65–6.47)
Parkinson's disease	0	0.16	−0.16	0.00	0.00–23.06	39	9.90	29.10	**3.94**	2.80–5.39	–
Overall	23	1.85	21.15	**12.43**	7.88–18.66	155	43.35	111.65	**3.57**	3.03–4.18	**3.47** (1.11–10.90)

Standardized Mortality Ratio (SMR) is standardized by age at death, calendar year of death, sex, and race. Excess deaths represent the observed deaths minus the expected deaths. Non-speed players are defensive and offensive linemen. CI = confidence interval, SRR = directly standardized rate ratio. Bold indicates p < 0.05.

## Discussion

Overall, NFL players had a higher rate of neurodegenerative mortality than the general population, particularly among speed players, those <60-years-old, and players with careers ≥5 seasons. Subtype-specific analyses revealed higher dementia, ALS, and PD mortality. Dementia and PD mortality were higher in players with careers ≥5 seasons, ALS mortality was higher among non-White players and those <60-years-old, and dementia mortality was higher in speed position players. These findings support our first and third hypotheses that NFL players demonstrate higher subtype-specific and overall neurodegenerative mortality but reduced all-cause mortality, although variations across position, race, career duration, and age were inconsistent, partially refuting our second hypothesis. Sensitivity analysis revealed neurogenerative mortality remained threefold higher after accounting for competing risks. Together, this study may provide reliable evidence of elevated neurodegenerative mortality among NFL players by overcoming key methodological limitations of prior work. Namely, the present study characterizes neurodegenerative mortality among a larger cohort throughout a longer follow-up period compared to prior studies.^2^^,^^3^

Our results demonstrate reduced overall mortality among NFL players but a pronounced risk of neurodegenerative mortality,^2^^,^^3^ supporting the link between RHI exposure and NDD. NFL players in speed positions had twice the dementia rate of non-speed players, potentially related to greater cumulative *g*-force exposure, which have been associated with higher NDD risk.^18^ Speed positions result in lower frequency but higher magnitude RHI,^19^ and record among the highest traumatic brain injury (TBI) rates per NFL game.^20^ The lack of positional differences in ALS or PD mortality is consistent with previous literature.^3^

Our results also reinforce a pattern of increasing neurodegenerative mortality risk with greater career duration,^18^^,^^21^ consistent with the well-established dose–response relationship between cumulative RHI exposure and NDD.^1^^,^^18^^,^^21^ By comparing within cohort relative differences, the observed elevation in neurodegenerative mortality among those with careers ≥5 seasons accounts for the selection and athletic resilience survivorship effects, and demonstrates that the relationship between cumulative RHI exposure and NDD remains despite this survival bias. Older individuals in the cohort are inherently more likely to develop NDDs due to their age.^1^^,^^18^ *LTASR* fundamentally accounts for potential age-related survivorship effects by comparing cohorts to an age-standardized general population with comparable age-related NDD risk. The robustness of the *LTASR* mortality risk estimates stems from its use of comprehensive stratified rate information, whereby each cohort member is compared with population rates standardized by sex, race, age, and calendar period.^14^

To address potential competing risks, whereby the survivorship effects observed among NFL players mechanistically increases the exposure to age-dependent outcomes such as NDD, a cause-specific hazard simulation was conducted. Competing risks alone would inflate the NDD SMR by a factor of 1.30, yielding a residual SMR of 3.04. These findings indicate that differential survivorship cannot account for the magnitude of the observed excess in neurodegenerative mortality, consistent with prior work in professional soccer players reporting that competing risks attenuate but do not eliminate the excess neurodegenerative mortality.^22^ Furthermore, tipping point analysis revealed that even under the biologically implausible assumption of zero mortality from any other competing cause, the residual neurodegenerative SMR remained statistically significant. Our results demonstrate that the excess in neurodegenerative mortality is not an artifact of elite athlete survivorship effects. Rather, age acts as a biological amplifier of an underlying vulnerability established by cumulative RHI exposure, consistent with broader literature suggesting that the pathological processes initiated by RHI progress and manifest clinically over decades following exposure.^1^^,^^23^ Together, these results reinforce a link between cumulative RHI exposure and NDD, underscoring the need for exposure mitigation strategies.^1^^,^^18^^,^^21^

The 15-fold increased risk of ALS and ∼8-fold risk of dementia mortality in players <60-years-old supports RHI exposure as a driver of earlier NDD pathogenesis.^1^^,^^18^^,^^21^ Notably, one of the three dementia decedents <60-years-old died in his forties, and before CTE was widely publicized.^24^ These results reinforce evidence of the hazards associated with American football and the link between cumulative RHI exposure and early-onset neurodegenerative mortality.^1^^,^^18^^,^^21^

The lower rate of ALS in the non-White general population relative to White individuals, coupled with substantial exposure during an NFL career, may amplify the relative ALS mortality risk observed in non-White players.^25^ Prior work on ALS mortality in NFL players similarly reported higher, although nonsignificant, ALS mortality in Black players compared to White players.^4^ Non-White players also died significantly younger than White counterparts, making up a disproportionate majority of ALS and dementia deaths <60-years-old. Non-White players also tended to have longer NFL careers and were more likely to play in speed positions, suggesting that increased exposure may confer greater risk of ALS and earlier onset ALS and dementia mortality among non-White players. Few investigations of NFL players explored racial differences in neurodegenerative disease.^4^^,^^26^ Of 152 young brain donors, those with CTE at death were more likely to be Black and older at death.^26^ Previous studies evaluating neurodegenerative mortality in NFL players lacked sufficient sample size to explore race- or age-based differences in mortality.^2^^,^^3^

The present data also reinforce an observed NDD-excluded potential survivorship effect,^3^ with lower rates of cancer, cardiovascular disease, injury, suicide, and all-cause mortality. The observed reduced risk of death by suicide is consistent with previous studies, which aligns with greater access to resources, higher resiliency, and lower rates of mental health conditions among NFL players.^27^ However, mental health remains a critical consideration for NFL players, influenced by factors such as adverse childhood experiences, lower socioeconomic status during childhood, and the stigma associated with seeking care.^28^ The broader observed NDD-excluded potential survivorship effect aligns with the NFL selection bias for physically fit individuals, as well as the benefits of regular exercise and medical care.^22^ Individuals who compete in elite level athletics are more likely to be physically and cognitively high-performing, behaviorally regulated, tolerant of high workloads, and less likely to smoke or have a major injury or disease that prevents exercise, compared to the general population.^29^ These unique differences may represent a variant of the healthy worker survivor effect. However, that terminology is not directly applicable, as elite athletes, particularly at the collegiate level, are not technically workers. In addition, given the extreme selection pressures of elite athletics participation, genetic and other factors likely play a larger role in determining who is able to participate compared to typical healthy workers.^29^ Based on these differences, a more appropriate name for this effect may be the selection through athletic resilience survivor (STARS) effect.

The NDD-excluded STARS effect among NFL players provides compelling evidence for RHI exposure-driven NDD pathogenesis, as the lower cardiovascular and other disease mortality would otherwise be expected to reduce intrinsic NDD risk.^30^ Although these contributing factors attribute to lower mortality rates in NFL players than the general population, these modifiable risk factors provide important targets for treatment plans for these individuals. Our results emphasize that this cohort should be treated as having higher risk for NDD, even in the absence of symptoms, given that a subset of these individuals are likely in the pre-clinical phase of NDD. As such, emphasized strategies should target modifiable risk factors of NDD to increase healthspan, or the period of life spent in good health, including education and prevention initiatives that combat mid-life cardiovascular and metabolic risk factors, such as hypertension, obesity, diabetes mellitus, hypercholesterolemia, and atherosclerosis.^31^ Throughout these athletes’ lifespan, prevention efforts should also focus on regular physical activity, mental health treatment, and maintaining social relationships.^31^ In addition to these critical modifiable factors, concerted multi-institutional efforts must work to reduce the cumulative RHI exposure sustained by American football players.

The elevated neurodegenerative mortality risk reported in our study underscores a crucial need to ensure NFL players receive appropriate care and therapeutic benefit from disease recognition.^32^ Furthermore, studies which aim to quantify the relationship between American football and NDD, including CTE, are critical to ensuring that individuals are able to take informed steps to reduce their risk.^31^

Our study had several limitations. Neurodegenerative mortality is frequently misclassified and this study risks underreporting its true prevalence.^33^ *LTASR* reports only White/non-White rates, preventing nuanced analyses of racial mortality differences. Without multiple cause of death rates, disease burden beyond underlying cause of death could not be evaluated. Given the size of the cohort and the relative rarity of dementia mortality <60-years-old, small changes in the number of cases could substantially alter these findings. Despite CTE's distinct neuropathology, it is frequently diagnosed as other NDDs in life and not recorded on death certificates, preventing determination of the proportion of decedents with CTE.^1^ Although this study identified early-onset neurodegenerative mortality, we lacked data on the age of NDD onset or diagnosis, precluding time to event analyses and estimates of disability from NDD, which are crucial areas for future work to inform interventions.

Despite the observed STARS effect, there may be some relative increases in cardiovascular disease among those with RHI exposure.^2^ Additionally, the STARS effect could increase NDD rates as players live longer; however, *LTASR's* robust age-adjustment mitigates this effect, and competing risks simulation reveal that it accounts for only a fraction of the excess. Similarly, there may be interactive effects between age and neurodegenerative disease risk, which may influence internal comparisons across career duration categories despite robust age adjustment. NFL participation, playing position, and career duration served as validated proxies of exposure but may not fully capture individual-level variation in cumulative impact burden. Death certificate data and LTASR precluded evaluation of subtype-specific mortality patterns in dementia. Other risk factors were unavailable for adjustment, including substance use, military experience, genetics, socioeconomic status, and comorbid conditions, representing an important area for future work. We cannot rule out that awareness of an individual's NFL career may have influenced death certificate reporting, though differential ascertainment bias is unlikely to be a source of error. Elevations in neurodegenerative disease may, in part, result from factors unrelated to exposure, including NFL selection pressures and unique genetic characteristics among NFL players. This study may not be generalizable to the broader American football community, applies only to males, and should be interpreted with appropriate context.

Neurodegenerative mortality was nearly four times higher than the general population in this fully enumerated cohort of NFL players, with subtype-specific elevations in dementia, ALS, and PD mortality. The present findings provided further support for an NDD-excluded STARS effect, with lower mortality observed for all other causes. Accounting for these survivorship benefits, NFL players had a threefold greater risk of neurodegenerative mortality than the general population. These results strongly support the increased risk of RHI exposure-related neurodegenerative mortality among NFL players that cannot be explained by differential survivorship, reinforcing the need for policies limiting RHI exposure and supporting evaluation of healthspan-focused interventions.

## Contributors

**CBL**: Conception or design of the work; accessed and verified data; acquisition, analysis, or interpretation of data for the work; drafting the work; read and approved the version to be published; agreement to be accountable for all aspects of the work in ensuring that questions related to the accuracy.

**BA**: Accessed and verified data; Acquisition, analysis, or interpretation of data for the work; reviewing it critically for important intellectual content; read and approved the version to be published; integrity of any part of the work are appropriately investigated and resolved.

**MJM**: Acquisition, analysis, or interpretation of data for the work; reviewing it critically for important intellectual content; read and approved the version to be published; integrity of any part of the work are appropriately investigated and resolved.

**CJN**: Acquisition, analysis, or interpretation of data for the work; reviewing it critically for important intellectual content; read and approved the version to be published; integrity of any part of the work are appropriately investigated and resolved.

**EDF**: Acquisition, analysis, or interpretation of data for the work; reviewing it critically for important intellectual content; read and approved the version to be published; integrity of any part of the work are appropriately investigated and resolved.

**BF**: Acquisition, analysis, or interpretation of data for the work; reviewing it critically for important intellectual content; read and approved the version to be published; integrity of any part of the work are appropriately investigated and resolved.

**AJW**: Acquisition, analysis, or interpretation of data for the work; reviewing it critically for important intellectual content; read and approved the version to be published; integrity of any part of the work are appropriately investigated and resolved.

**EJC**: Acquisition, analysis, or interpretation of data for the work; reviewing it critically for important intellectual content; read and approved the version to be published; integrity of any part of the work are appropriately investigated and resolved.

**CAR**: Acquisition, analysis, or interpretation of data for the work; reviewing it critically for important intellectual content; read and approved the version to be published; integrity of any part of the work are appropriately investigated and resolved.

**RDZ**: Acquisition, analysis, or interpretation of data for the work; reviewing it critically for important intellectual content; read and approved the version to be published; integrity of any part of the work are appropriately investigated and resolved.

**MLA**: Conception or design of the work; acquisition, analysis, or interpretation of data for the work; reviewing it critically for important intellectual content; read and approved the version to be published; integrity of any part of the work are appropriately investigated and resolved.

**ACM**: Conception or design of the work; acquisition, analysis, or interpretation of data for the work; reviewing it critically for important intellectual content; read and approved the version to be published; integrity of any part of the work are appropriately investigated and resolved.

**JM**: Conception or design of the work; acquisition, analysis, or interpretation of data for the work; reviewing it critically for important intellectual content; read and approved the version to be published; agreement to be accountable for all aspects of the work in ensuring that questions related to the accuracy.

**DHD**: Conception or design of the work; accessed and verified data; acquisition, analysis, or interpretation of data for the work; reviewing it critically for important intellectual content; read and approved the version to be published; agreement to be accountable for all aspects of the work in ensuring that questions related to the accuracy.

## Data sharing statement

Data available upon appropriate request and initiation of a data use agreement.

## Declaration of interests

CBL reports receiving clinical funding from the Brain and Body Program funded by the NFLPA outside the submitted work. MJM reports receiving clinical funding from the Brain and Body Program funded by the NFLPA outside the submitted work. CJN reported serving as a volunteer member of the Mackey-White Committee of the National Football League (NFL) Players Association, for which he receives travel support, and an advisor and options-holder with Oxeia Biopharmaceuticals, LLC, and StataDx; receiving travel support from the NFL, NFL Players Association, World Rugby, World Wrestling Entertainment (WWE), and All Elite Wrestling (AEW); serving as an expert witness in cases related to concussion and chronic traumatic encephalopathy (CTE) and receiving compensation for speaking appearances and serving on the Players Advocacy Committee for the NFL Concussion Settlement; and being employed by the Concussion and CTE Foundation, a 501(c) (3) nonprofit which receives charitable donations. AJW is employed by the Professional Footballers’ Association, the trade union for professional soccer players in England. RDZ reported royalties from Springer/Demos Publishing for serving as co-editor of the text Brain Injury Medicine; serving on the scientific advisory board of Myomo, onecare.ai, Kisbee, and NanoDiagnostics; being partially supported by the National Institute on Disability, Independent Living, and Rehabilitation Research; and funding from the Football Players Health Study at Harvard University, which is funded by the NFLPA. MLA reported receiving grants from Life Molecular Imaging and Rainwater Charitable Foundation and personal fees from Oxford University Press and Michael J Fox Foundation outside the submitted work. ACM reported serving as a member of the Mackey-White Committee of the National Football League Players Association. JM reported receiving grants from the NIH outside the submitted work. DHD reported receiving personal fees for providing expert testimony related to traumatic brain injury and spinal cord injury, serving as a medical advisor and options holder for StataDx, receiving research funding from the Football Players Health Study at Harvard University (FPHS) funded by the NFL Players Association (NFLPA), serving as a volunteer member of the Mackey-White Committee of the NFLPA, and receiving clinical funding from the Brain and Body Program funded by the NFLPA, all outside the submitted work.
