# Supplementary material for: Neurodegenerative mortality among National Football League Players
Source: eClinicalMedicine. 2026 Jul 8;97:104051. doi: 10.1016/j.eclinm.2026.104051 (PMC13420610; doi:10.1016/j.eclinm.2026.104051)
Supplement: Supplementary Tables [file mmc1.docx]

**Neurodegenerative Mortality Among National Football League Players**

**Authors:** Charlotte B. Luster, B.A.; Bobak Abdolmohammadi, B.A.; Michael J. Mastrodicasa, B.S.; Christopher J. Nowinski, Ph.D.; Evan D. Feigel, Ph.D.; Brenna Finegan, M.P.H.; Adam J. White, Ph.D.; Eric J. Connors, Ph.D.; Craig Rovito, M.D.; Ross D. Zafonte, D.O; Michael L. Alosco, Ph.D.; Ann C. McKee, M.D.; Jesse Mez, M.D., M.S. Daniel H. Daneshvar, M.D., Ph.D.

**Supplementary Material**

Supplemental Table 1. LTASR Neurodegenerative Disease International Classification of Diseases (ICD) Code Mapping

Supplemental Table 2. Racial Composition of National Football League Decedents by Age at Death

Supplemental Table 3. Sample Distribution by Age Group

Supplemental Table 4. Age Differences in Neurodegenerative Mortality in National Football League Players

| **Supplemental Table 1. LTASR Neurodegenerative Disease International Classification of Diseases (ICD) Code Mapping** | |
| --- | --- |
| Underlying Cause of Death | Code Mapping |
| Amyotrophic Lateral Sclerosis | ICD-9: 335.2, 335.21, 335.24, 335.29  ICD-10: G12.2 |
| All-cause Dementia | ICD-9: 290, 290.0, 290.1, 290.2, 290.3, 290.4, 290.43, 290.9, 294.2, 331, 331.0, 331.2  ICD-10: F01, F01.0, F01.1, F01.2, F01.3, F01.8, F01.9, F03, G30, G30.0, G30.1, G30.8, G30.9, G31.1 |
| Parkinson's Disease | ICD-9: 332, 332.0 332.1  ICD-10: G20, G21, G21.0, G21.1, G21.2, G21.3, G21.8, G21.9 |
| International Classification of Diseases, 9th Revision (ICD-9) and International Classification of Diseases, 10th Revision (ICD-10) codes within each neurodegenerative disease classification. LTASR = Life Table Analysis System R package | |

| **Supplemental Table 2. Racial Composition of National Football League Decedents by Age at Death** | | | | |
| --- | --- | --- | --- | --- |
| ALS Decedents | | | | |
| Race | Under 50 years old at Death | 50+ years old at Death | Under 60 years old at Death | 60+ years old at Death |
| Non-White | 7 (46.7%) | 8 (53.3%) | 11 (73.3%) | 4 (26.7%) |
| White | 3 (16.7%) | 15 (83.3%) | 9 (50.0%) | 9 (50.0%) |
| Dementia Decedents | | | | |
| Race | Under 50 years old at Death | 50+ years old at Death | Under 60 years old at Death | 60+ years old at Death |
| Non-White | 1 (3.4%) | 28 (96.6%) | 2 (6.9%) | 27 (93.1%) |
| White | 0 (0%) | 77 (100%) | 1 (1.3) | 76 (98.7%) |
| Percentages represent the proportion within each racial group. | | | | |

| **Supplemental Table 3. Sample Distribution by Age Group** | |
| --- | --- |
| Age Group | Sample Size |
| 20-25 | 22 |
| 25-30 | 85 |
| 30-35 | 55 |
| 35-40 | 101 |
| 40-45 | 123 |
| 45-50 | 131 |
| 50-55 | 158 |
| 55-60 | 182 |
| 60-65 | 233 |
| 65-70 | 251 |
| 70-75 | 283 |
| 75-80 | 231 |
| 80-85 | 159 |
| 85-90 | 7 |
| 90-95 | 1 |
| Age groups exclude the upper bound. | |

| **Supplemental Table 4. Age Differences in Neurodegenerative Mortality in National Football League Players** | | | | | | | | | |
| --- | --- | --- | --- | --- | --- | --- | --- | --- | --- |
|  | Under Age 50 at Death | | | | Age 50+ at Death | | | | Under vs Over 50: SRR (95% CI) |
| Underlying Cause of Death | Observed | Expected | SMR | 95% CI | Observed | Expected | SMR | 95% CI |  |
| ALS | 10 | 0.24 | **41.67** | 19.98-76.63 | 23 | 7.02 | **3.28** | 2.08-4.92 | **12.72** (6.05-26.72) |
| All-cause Dementia | 1 | 0.03 | 33.33 | 0.84-185.72 | 105 | 27.85 | **3.77** | 2.08-4.56 | **8.84** (1.23-63.35) |
| Parkinson's Disease | 0 | 0.02 | 0.00 | 0.00-184.44 | 39 | 10.03 | **3.89** | 2.76-5.32 | - |
| Overall | 11 | 0.29 | **37.93** | 18.94-67.87 | 167 | 44.90 | **3.71** | 2.76-5.32 | **10.20** (5.54-18.77) |
| Standardized Mortality Ratio (SMR) is standardized by age at death, calendar year of death, sex, and race. CI = confidence interval, SRR = directly standardized rate ratio | | | | | | | | | |
